# A Nested Case-Control Study of Intrauterine Exposure to Persistent Organochlorine Pollutants in Relation to Risk of Type 1 Diabetes

**DOI:** 10.1371/journal.pone.0011281

**Published:** 2010-06-23

**Authors:** Anna Rignell-Hydbom, Maria Elfving, Sten A. Ivarsson, Christian Lindh, Bo A. G. Jönsson, Per Olofsson, Lars Rylander

**Affiliations:** 1 Division of Occupational and Environmental Medicine, Lund University, Lund, Sweden; 2 Department of Pediatrics, Lund University Hospital, Lund University, Lund, Sweden; 3 Department of Clinical Sciences, Unit of Pediatric Endocrinology, Lund University/CRC, Malmö, Sweden; 4 Department of Obstetrics and Gynecology, Malmö University Hospital, Lund University, Malmö, Sweden; East Carolina University, United States of America

## Abstract

**Background:**

The incidence of type 1 diabetes in Europe is increasing at a rate of about 3% per year and there is also an increasing incidence throughout the world. Type 1 diabetes is a complex disease caused by multiple genetic and environmental factors. Persistent organochlorine pollutants (POPs) have been suggested as a triggering factor for developing childhood type 1 diabetes. The aim of this case-control study was to assess possible impacts of *in utero* exposure to POPs on type 1 diabetes.

**Methodology/ Principal Findings:**

The study was performed as a case-control study within a biobank in Malmö, a city located in the Southern part of Sweden. The study included 150 cases (children who had their diagnosis mostly before 18 years of age) and 150 controls, matched for gender and day of birth. 2,2′,4,4′,5,5′-hexachlorobiphenyl (PCB-153) and the major DDT metabolite 1,1-dichloro-2,2-bis (*p*-chlorophenyl)-ethylene (*p,p′*-DDE) were used as a biomarkers for POP exposure. When comparing the quartile with the highest maternal serum concentrations of PCB-153 with the other quartiles, an odds ratio (OR) of 0.73 (95% confidence interval [CI] 0.42, 1.27) was obtained. Similar results was obtained for *p,p′*-DDE (OR 0.56, 95% CI 0.29, 1.08).

**Conclusions:**

The hypothesis that *in utero* exposure to POPs will trigger the risk for developing type 1 diabetes was not supported by the results. The risk estimates did, although not statistically significant, go in the opposite direction. However, it is not reasonable to believe that exposure to POPs should protect against type 1 diabetes.

## Introduction

The incidence of type 1 diabetes in Europe and many other parts of the world is increasing at a rate of about 3% per year [1]–[5]. The increase is most pronounced among children in the age-group of 0–4 years particularly in the high-incidence populations of Sweden, Finland and UK [5]–[7]. Type 1 diabetes is an autoimmune disease that is characterized by destruction of insulin-producing pancreatic β-cells, resulting in a lack of or even absence of insulin production and disturbed glucose homeostasis [8]. It is a complex disease caused by multiple genetic and environmental factors. It is evident that genetic factors play an important role and the Human Leukocyte Antigen (HLA) class II genotype on chromosome 6 is most strongly associated with an increased risk [9]. However, children with high risk HLA susceptibility generally progress to clinical disease in less than 10% [10]. The rapid increase and the wide spectrum of HLA types among children with diabetes suggest that life-style or environmental factors play a role in the pathogenesis. In addition, the increasing incidence in populations moving from low to high risk areas indicates that life-style or environmental modification is part of the etiology [11]. Several studies support the hypothesis that prenatal exposure to environmental factors are of importance. In this context, advanced maternal age, blood group incompatibility between mother and child [12], maternal enterovirus infections during pregnancy [13]–[14] and being born with congenital rubella [15] are associated with an increased risk of developing the disease later in life.

Among environmental pollutants suggested to trigger development of type 1 diabetes in childhood is persistent organochlorine pollutants (POPs), such as polychlorinated biphenyls (PCBs), dioxins and the pesticide DDT. Circumstantial evidence support that *in utero* exposure to POPs, have a triggering mechanism for childhood diabetes. Both *in vitro* and *in vivo* studies in mice, rats and guinea pigs exposed to the most toxic dioxin compound, 2,3,7,8-tetrachlorodibenzo-*p*-dioxin (TCDD), have been linked to drastic reductions in cellular glucose uptake [16], and in rabbits also a lowering of the insulin production [17]. There is also a biologic plausibility for a link between dioxin-like compounds and diabetes through interaction between aryl hydrocarbon receptor and peroxisome proliferator-activated receptor mediated signaling pathways [18]–[21]. Moreover, studies in mice predisposed to development of autoimmune disease have shown that administration of TCDD during gestation leads to exacerbated postnatal autoimmunity [22]. In general, the doses used in animal studies are higher as compared to the background levels that humans are exposed to. On the other hand, the general population is most often continuously exposed. However, there is only one epidemiological study addressing the association between POP and type 1 diabetes. In a small study on pregnant women, serum levels of PCBs were 30% higher among those women with diabetes (mostly type 1 diabetes) than among those without [23]. Since the study was cross-sectional, the causal relationship could not be determined.

POPs have been released in our environment, since World War II. These pollutants are accumulated particularly in highly rank predators in the aquatic food chain. In Sweden, consumption of fatty fish from the Baltic Sea is the major source of POPs, but also dairy products and meat, contain these pollutants [24].

The aim of this case-control study was to investigate, by comparing stored serum samples from delivery, if there is an association between *in utero* exposure to POPs and risk for the child to develop type 1 diabetes.

## Materials and Methods

### Study population and design

The vast majority of deliveries in the city of Malmö, a city with around 250.000 inhabitants situated in southern Sweden, take place at the Malmö University Hospital maternity unit. Since 1970, venous blood samples from mothers and umbilical cord blood samples from the newborns have routinely been collected. Serum samples from approximately 70,000 deliveries are stored frozen at −20C° and cases and controls for this study were selected from this serum bank. The study was performed in accordance with the Declaration of Helsinki and approved by the research Ethics Committee at Lund University. All participants gave their verbal informed consent at the time blood samples were collected. From the1960th to the 1990th written informed consent was not used, at this time only verbal informed consent was used. The women who were willing to donate a blood sample for research were informed that the sample in the future could be used for research. When this study took place the Ethic committee approved the study and in addition we informed about the study in the local newspaper and in this advertisement we gave the women who ones had approved that their sample could be used for research an opportunity to change their mind and by a phone call tell us that the sample should be destroyed. The data were analyzed anonymously.

### Cases

Among children born from February 1970 to December 1990, 150 (82 boys and 68 girls, Table 1) have developed type 1 diabetes before year 2002. The vast majority (88%) of these cases was diagnosed before the age of 18 years and almost all were diagnosed and treated at the Department of Paediatrics at Malmö University Hospital. Patients who developed type 1 diabetes between 18 and 27 years were identified using the Diabetes Incidence Study in Sweden (DISS) registry [25]. Five cases had a mother with type 1 diabetes (3%) and 8 a father with type 1 diabetes (5%).

**Table 1:** Background characteristics among 150 children from the Southern part of Sweden who developed type 1 diabetes (cases) and 150 matched controls.

	Cases		Controls	
	n	(%)	n	(%)
Birth year^a^				
1970–1975	47	(31)	45	(30)
1976–1980	26	(17)	28	(19)
1981–1985	36	(24)	36	(24)
1986–1990	41	(27)	41	(27)
Males^a^	82	(55)	-	
Age at diagnosis (years)				
<5	17	(11)	-	
5–9	43	(29)	-	
10–17	72	(48)	-	
≥18	18	(12)	-	
Maternal age (years)				
<20	6	(4.0)	9	(6.0)
20–24	40	(27)	33	(22)
25–29	57	(38)	65	(43)
30–34	33	(22)	28	(19)
>35	14	(9.3)	15	(10)
Primiparous, i.e. parity = 1^b^	66	(52)	57	(45)
Maternal smoking in early pregnancy^c^	10	(18)	15	(28)
Preterm (<37 weeks)^d^	6	(4.7)	6	(4.8)
Birth weight <2500 g^e^	1	(0.8)	4	(3.2)
Birth weight >4500 g	8	(6.3)	3	(2.4)

a Matching variable.

b Data missing for 22 cases and 24 controls.

c Data missing for 93 cases and 96 controls (data only available from 1983 and onwards).

d Data missing for 22 cases and 24 controls.

e Data missing for 22 cases and 26 controls.

### Controls

For each case, a non-diabetic control was selected from the serum bank and matched for gender and date of birth (with the exception for two controls the differences were ±9 months).

### Biomarkers of exposure

In the present study 2,2′,4,4′,5,5′-hexachlorobiphenyl (PCB-153) has been used as a biomarker for POP exposure since PCB-153 correlates very well (r = 0.98) with total PCB concentrations in plasma and serum from Swedish individuals [26]–[27], with the TCDD equivalent (TEQ) in plasma from PCB (r = 0.89). The major DDT metabolite 1,1-dichloro-2,2-bis (*p*-chlorophenyl)-ethylene (*p,p*′-DDE), which is detectable in a high proportion of adult Swedes, was also used as a biomarker for POP exposure [28].

### Chemical analyses

PCB-153 and *p,p*′-DDE were analyzed as described in Rignell-Hydbom et al. [29]. The compounds were extracted from 0.05 mL aliquots of serum by solid phase extraction (Isolute ENV+; IST, Hengoed, UK) using on-column degradation of the lipids and analysis by gas chromatography mass spectrometry. ^13^C_12_-labeled PCB-153 and ^13^C_12_-labeled *p,p*′-DDE were used as internal standards. The relative Coefficient of Variation (CV), calculated from 330 samples analyzed in duplicate at different days, were 10% at 3 ng/mL for PCB-153 and 12% at 13 ng/mL for *p,p*′-DDE. The quantification limits were 0.05 ng/mL for PCB-153 and 0.1 ng/mL for *p,p*′-DDE. The analyses were part of a round-robin inter-comparison program (University of Erlangen-Nuremberg, Germany) and our results were within the tolerance limits.

### Additional data collection

By linkage to the Swedish Medical Birth Register information was obtained about parity, gestational length and birth weight (Table 1). Information on maternal smoking in early pregnancy was only available from 1983 and onwards, and was accordingly missing for most of the material (63%).

### Statistical analyses

By use of the sign test [30], we investigated whether the POP exposure levels differed between the cases and the controls in a systematic way. In addition, we performed separate analyses for the children born between 1970 and 1980, and for the children born between 1981 and 1990.

The association between the absolute levels of maternal POP concentrations and the risk in the offspring to develop type 1 diabetes was evaluated by conditional logistic regression (using the statistical software EGRET), given odds ratios (OR) as the risk measure with 95% confidence intervals (CI). The exposure variables (PCB-153 and *p,p*′-DDE) were categorized into quartiles based on the distributions among controls. The lowest exposure category was the reference category. We did also estimate p-values for trend by including the categorized exposure variables as continuous variables (coded 0, 1, 2 and 3) in the models. Due to the relatively high correlation between PCB-153 and *p,p′*-DDE (r_s_ = 0.63) concentrations we did not include these exposure measures simultaneously in the models. We did, however, create a trichotomized exposure variable based on PCB-153 and *p,p*′-DDE concentrations. The three categories was defined as i) children whose mothers had exposure levels of PCB-153 in the lowest quartile and *p,p′*-DDE in the lowest quartile, ii) children whose mothers had exposure levels of PCB-153 in highest quartile and *p,p′*-DDE in the highest quartile, and iii) all other. Maternal age, parity, preterm birth (<37 weeks), high birth weight (<4500 g), and smoking habits in early pregnancy were all considered as potential confounders.

We compared the exposure levels between the cases whom had their type 1 diabetes diagnosed before the age of 5 years and the cases who developed type 1 diabetes diagnosed after the age of 18 years with Mann-Whitney test. By definition, children who developed type 1 diabetes after the age of 18 years had to be born before 1984, and in this latter comparison we only included children born before 1984.

## Results

### Exposure levels

**Figure 1:**
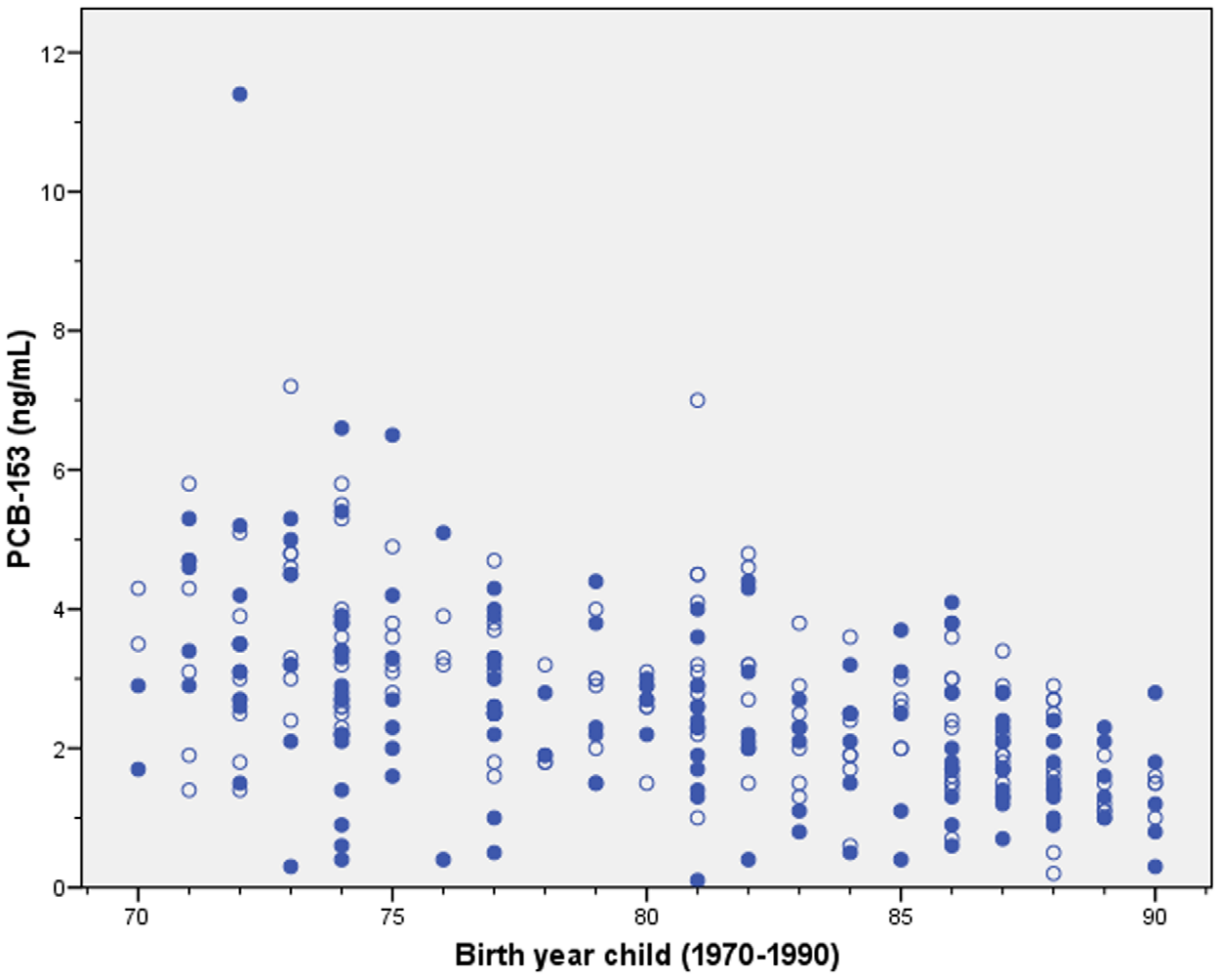
The median maternal serum concentrations of PCB-153 from 1970–1990.

The median maternal serum concentration of PCB-153 was 2.4 ng/mL (range 0.1, 11.4) in cases and 2.6 ng/mL (0.2, 7.2) in controls. Among all children the exposure levels were significantly (p<0.001) decreased over the study period (r_s_ = −0.47 and r_s_ = −0.54, respectively; Figure 1).

**Figure 2:**
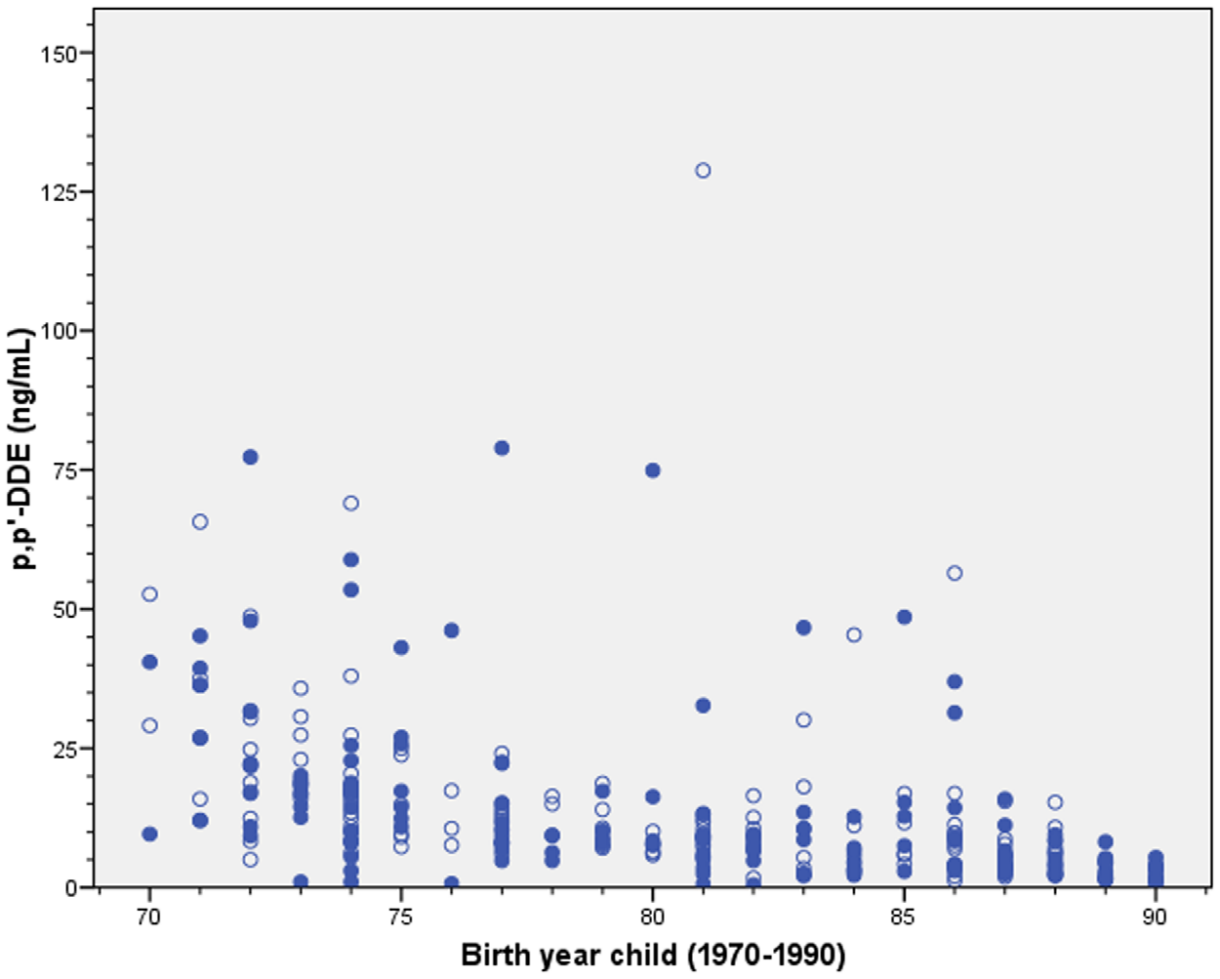
The median maternal serum concentrations of *p,p′*-DDE from 1970–1990.

The median maternal serum concentrations of *p,p′*-DDE were 9.2 ng/mL (0.5, 79) in cases and 9.6 ng/mL (0.9, 129) in controls. As for PCB-153, the levels of *p,p′*-DDE decreased over time (r_s_ = −0.60 and r_s_ = −0.68, respectively; Figure 2).

### PCB-153 and type 1 diabetes

In the present study, all potential confounders showed very weak and non-significant (all p-values >0.3) associations with type 1 diabetes. In addition, the distributions of these variables among cases and controls were similar (Table 1). Accordingly, except for the matching variables (gender and date of birth) we did not adjust for additional variables in the conditional logistic regression analyses.

When comparing the group with the highest maternal serum concentrations of PCB-153 with the group with the lowest concentrations, an OR of 0.64 (95% CI 0.32, 1.29) was obtained (Table 2). The upper three exposure quartiles gave ORs below unit i.e 1.0, although not statistically significant, when they were compared with the lowest exposure quartile. In addition, there were no statistically significant trend (p = 0.17). When the upper three exposure quartiles were merged into one group, and compared with the lowest exposure quartile, an OR of 0.73 (95% CI 0.42, 1.27) was obtained, corresponding to a p-value of 0.27. The ORs were very similar when the analyses included only male children (data not shown).

**Table 2:** Maternal serum concentrations of PCB-153 and *p,p′*-DDE at delivery, and the risk for developing type 1 diabetes among their infants.

	Cases	Controls	OR	95% CI	p for trend
PCB-153 (ng/mL)					0.17
<1.9 (ref)	47	39	1.00	-	
1.9–2.6	39	36	0.85	0.45, 1.63	
2.7–3.4	33	38	0.67	0.35, 1.31	
>3.4	31	37	0.64	0.32, 1.29	
*p,p′*-DDE (ng/mL)					0.29
<5.8 (ref)	47	36	1.00	-	
5.8–9.6	36	40	0.58	0.28, 1.20	
9.7–16.8	28	37	0.51	0.24, 1.07	
>16.8	39	37	0.64	0.28, 1.46	

Odds ratios (OR) and 95% confidence intervals (CI) were obtained with conditional logistic regression analysis.

Out of the 150 cases, 85 had lower PCB-153 levels as compared to their matched control, given a p-value of 0.07. There were no systematic difference (p = 0.47) for the early period (born 1980 or earlier), whereas there was a statistically significant (p = 0.04) difference for the latter period (born 1981 or later), where 48 out of the 77 cases had lower levels of PCB-153 as compared to their matched control.

### *p,p′*-DDE and type 1 diabetes

As was observed for PCB-153, the upper three exposure quartiles did all give ORs below unit when they were compared with the lowest exposure quartiles (Table 2). However, there were no statistically significant trend (p = 0.29). When the upper three exposure quartiles were merged into one group, and compared with the lowest exposure quartile, an OR of 0.56 (95% CI 0.29, 1.08) was obtained, corresponding to a p-value of 0.08. The ORs were very similar when the analyses included only male children (data not shown).

For *p,p′*-DDE exposure there were no systematic differences between cases and controls when paired comparisons (by sign test) were performed, irrespectively of study period investigated.

### PCB-153 and *p,p′*-DDE and type 1 diabetes

The children whose mothers were categorized into the upper PCB-153 quartile as well as in the upper *p,p′*-DDE quartile had a non-significant decreased risk (OR 0.50, 95% CI 0.20, 1.25) of developing type 1 diabetes as compared to children whose mothers were categorized into the lowest PCB-153 quartile as well as in the lowest *p,p′*-DDE quartile

### Age at type 1 diabetes

The maternal levels of PCB-153 and *p,p′*-DDE did not differ (p = 0.80 and p = 0.59, respectively) between the mothers whose children had their type 1 diabetes diagnosed before the age of five years (n = 10) and the mothers whose children had their diabetes after the age of 18 years (n = 18).

## Discussion

This study did not show what we expected; a higher risk of type 1 diabetes in the offspring when the woman was exposed to POPs during pregnancy. The hypothesis that *in utero* exposure to POPs will trigger a development of type 1 diabetes was not supported by our data. Actually, the trend was in the opposite direction; with a higher POPs concentration in maternal serum, the risk of diabetes in the offspring was lower. This raises the question whether exposure to POPs have a protective effect. However, we suggest an alternative explanation. In Sweden, fish intake is associated with higher levels of POP biomarkers in blood [31]. These biomarkers might, however, not only be a proxy for the toxic compounds, but also for the essential long-chain fatty acids eicosapentaenoic acid (EPA) and docosahexaenoic acid (DHA) present in fatty fish [32]. EPA and DHA are incorporated in the cell membranes and have anti-inflammatory effects that might protect against type 1 diabetes. A Norwegian study, comprising 545 children with type 1 diabetes and 1668 control subjects, found a significant relationship between intake of cod-liver oil and a decreased risk of type 1 diabetes, possibly through the effects of long-chain n-3 fatty acids [33]. Thus one might speculate that PCB-153 as well as *p,p′*-DDE might act as indicators for food intake of polyunsaturated fatty acids (PUFA), which might protect against the risk of developing type 1 diabetes. The concentrations of POPs, especially PCB and DDT, in fatty fish caught around the Swedish coast decreased during the current study period [34]. In the present study there was a decrease regarding serum POP concentrations in maternal serum, which implies that the ratio between toxic compounds and beneficial PUFAs might have changed over time.

The foetal period is a vulnerable period for toxic substances [35]. POPs can pass the placental barrier [36] and most POPs have very long half-lives, five to ten years [36]. It is then reasonable to believe that POP levels measured in serum women reflect the *in utero* exposure in the fetus.

Due to the limited amount of serum available from the biobank, the chemical analyses were restricted to CB-153 and *p,p′*-DDE and we had no possibility to lipid adjust our samples. However, we do not see this as a major problem due to the very strong correlations (r>0.90) found between fresh and lipid adjusted samples [27].

The access to stored serum samples collected at delivery within a well-defined cohort is a major strength in the present study. There are no reasons to believe that there is a selection due to exposure. Moreover, by linkage to the Swedish Medical Birth Register, which covers about 97–98% of all births in Sweden since 1973, we had the possibility to collect information about possible confounders. The study did accordingly not lack information of known strong potential confounders, although data for smoking habits were missing for the majority of the participants. However, we can not exclude that other factors created some statistical noise, thereby biasing the results.

In conclusion, the results from the present study did not support POP exposure to be a triggering factor to develop type 1 diabetes. The risk estimates went in the opposite direction. In future studies we recommend that biomarkers for food consumption, such as PUFAs, should be analyzed to investigate the relation between PUFAs and the risk of developing islet autoimmunity or type 1 diabetes among children.
